# Supplementary material for: Helical Hybrid Nanostructure Based on Chiral M13 Bacteriophage via Evaporation-Induced Three-Dimensional Process
Source: Nanomaterials (Basel). 2024 Jul 16;14(14):1208. doi: 10.3390/nano14141208 (PMC11280118; doi:10.3390/nano14141208)
Supplement: Supplementary file 1 [file nanomaterials-14-01208-s001.zip › nanomaterials-3081905-supplementary.pdf]

Supporting Information

Helical Hybrid Nanostructure Based on Chiral M13 Bacteriophage via Evaporation-Induced Three-Dimensional Process

Thanh Mien Nguyen [1,2,†], Sung-Jo Kim [2,†], Dae Gon Ryu [3,†], Jae Hun Chung [4], Si-Hak Lee [4], Sun-Hwi Hwang [4], Cheol Woong Choi [3,*] and Jin-Woo Oh [1,2,5,*]

[1] BK21 FOUR Education and Research Division for Energy Convergence Technology, Pusan National
University, Busan 46241, Republic of Korea; ntmien93@pusan.ac.kr
[2] Institute of Nanobio Convergence, Pusan National University, Busan 46241, Republic of Korea;
sungjokim84@pusan.ac.kr
[3] Department of Internal Medicine, Medical Research Institute, Pusan National University School of Medicine and Research Institute for Convergence of Biomedical Science and Technology, Pusan National University Yangsan Hospital, Yangsan 50612, Republic of Korea; gon22gon@naver.com
[4] Department of Surgery, Pusan National University School of Medicine and Research Institute for
Convergence of Biomedical Science and Technology, Pusan National University Yangsan Hospital,
Yangsan 50612, Republic of Korea; jhchung@pnuyh.co.kr (J.H.C.); ghost109@hanmail.net (S.-H.L.); shhwang@pnuyh.co.kr (S.-H.H.)
[5] Department of Nanoenergy Engineering and Research Center for Energy Convergence Technology, Pusan National University, Busan 46241, Republic of Korea
* Correspondence: drluckyace@pusan.ac.kr (C.W.C.), ojw@pusan.ac.kr (J.-W.O.)
† These authors contributed equally to this work.

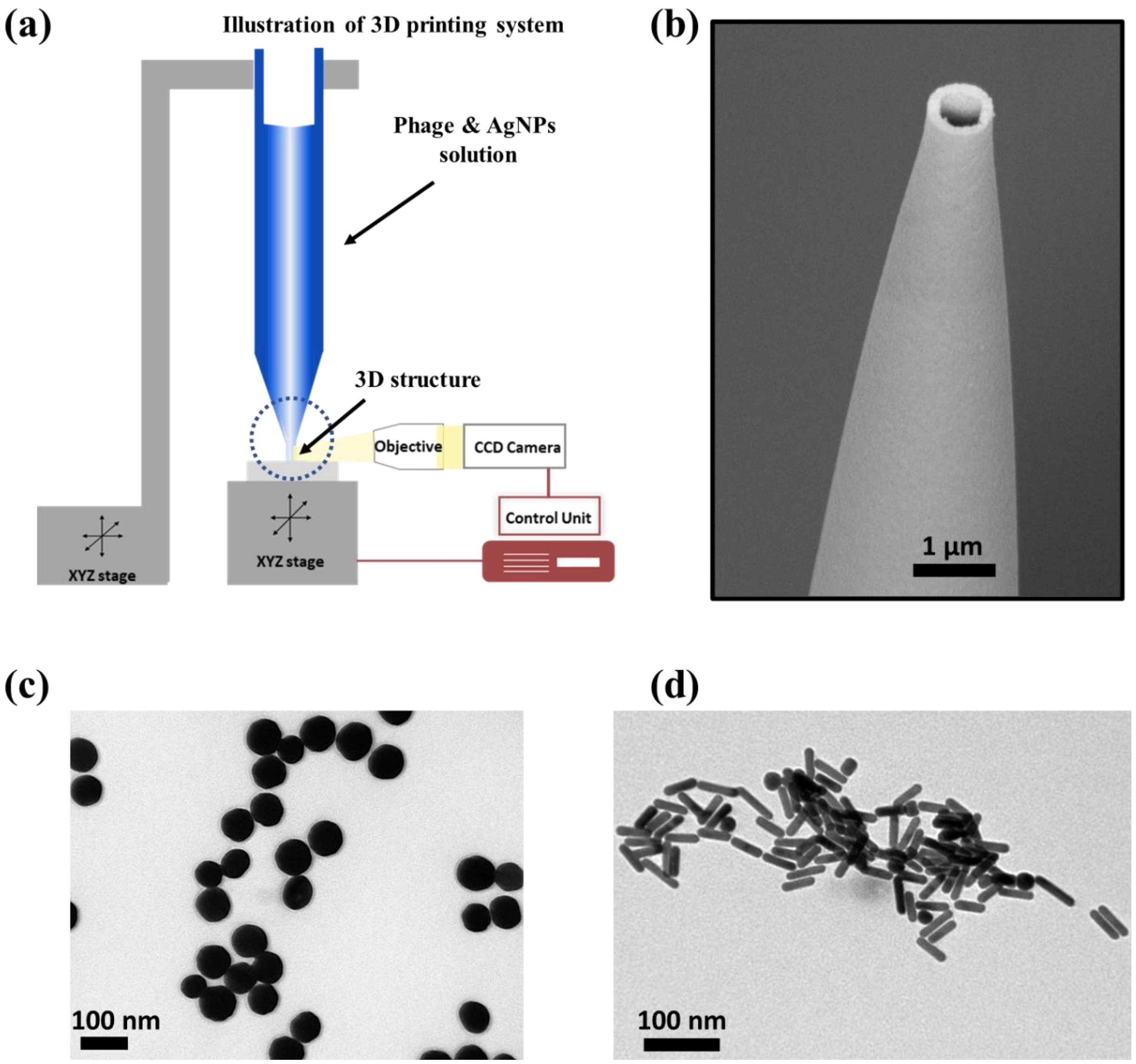

Figure S1: (a) Schematic illustration of 3D printing system with hybrid ink-based nanoparticles and bacteriophage. (b) SEM image of nanopipette with approximately 600 nm inner diameter. (c-d) TEM images of Ag nanoparticle 75 nm (c), gold nanorod with lengths and diameters of 55 nm and 15 nm.

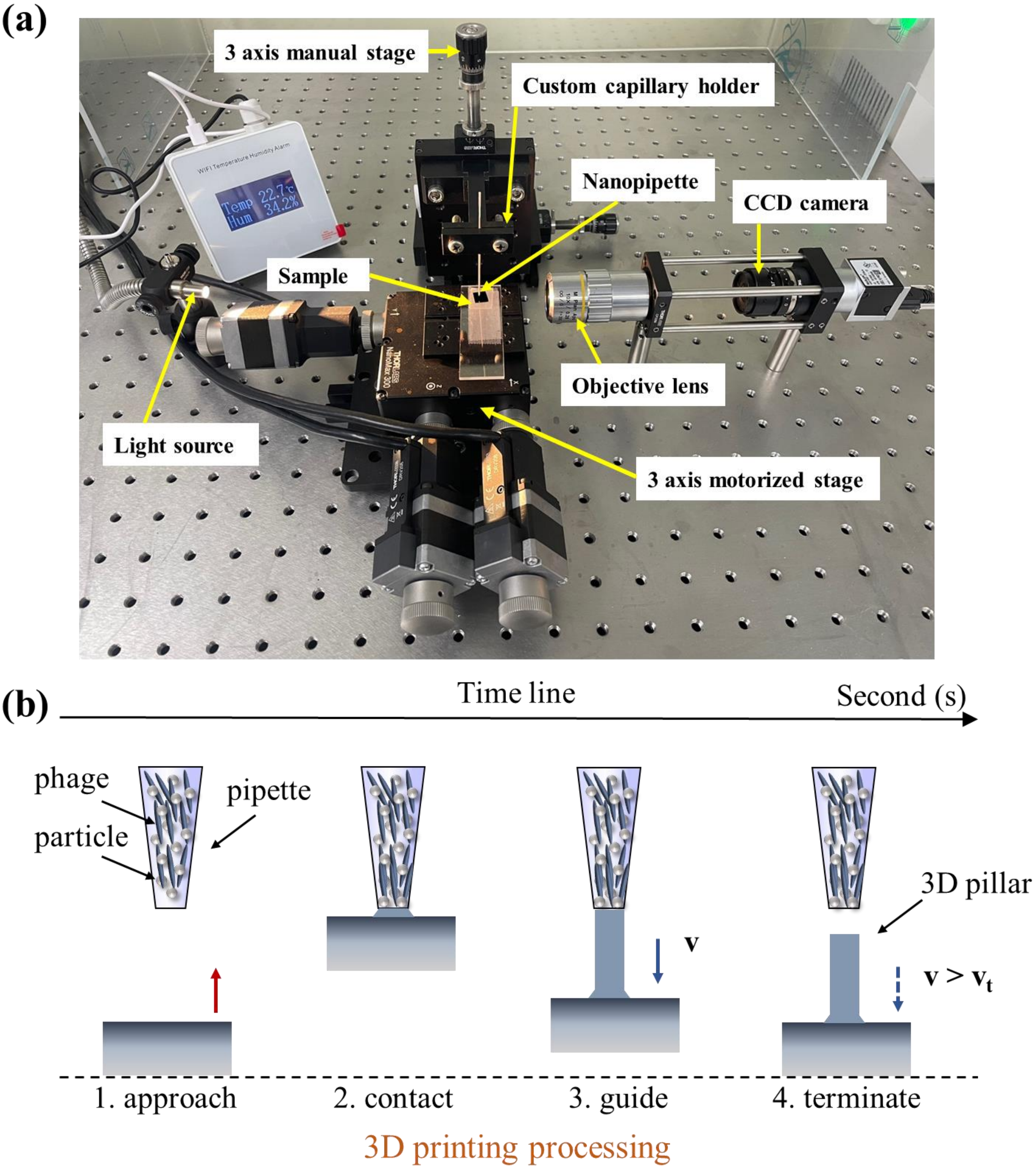

Figure S2: (a) Photograph of home-built 3D printing system. (b) 3D printing process including four steps: approach, contact, guide, and terminate.
